# Supplementary material for: Halogen-Free Flame Retardant Polypropylene Fibers with Modified Intumescent Flame Retardant: Preparation, Characterization, Properties and Mode of Action
Source: Polymers (Basel). 2021 Jul 31;13(15):2553. doi: 10.3390/polym13152553 (PMC8347993; doi:10.3390/polym13152553)
Supplement: Supplementary file 1 [file polymers-13-02553-s001.zip › polymers-1299876-supplementary.pdf]

Supporting Information:

Halogen-free flame retardant polypropylene fibers with modified intumescent flame retardant: preparation, characterization, properties and mechanism

Qibin Xu[1], Lei Wu[1], Xiang Yan[1], Shengchang Zhang[1], Linan Dong[2], Zexi Su[1], Tianhaoyue Zhong[1], Chunhui Jiang[1] , Yuan Chen[1], Mengjin Jiang[1], Pengqing Liu[1,*]

1. College of Polymer Science & Engineering, Sichuan University, Chengdu 610065, China
2. Chongqing Academy of Metrology and Quality Inspection, Chongqing 401120, China

* Corresponding author, Email: liupq@scu.edu.cn; Tel: (86)-28-8546 2013;

1. Pretreatment analysis of the flame retardant

The coupling agent KH-550 was used to perform surface treatment on the flame retardant in a high-speed pulverizer. As shown in SEM images of Dohor-6000A (Figure S1a) and modified Dohor-6000A (Figure S1b), the agglomeration of the flame retardant is significantly reduced after modification. The curves of the particle size distribution before and after modification are shown in Figure S1c and the related data is given in Table S1†. The particle size curve of the modified Dohor-6000A shifted to the left and changed from a single peak to a double peak, indicating that a part of the particles with larger size were transformed into particles with smaller size. The average volume particles size of the modified Dohor-6000A is reduced from 7.79 μm to 6.48 μm. This is because the flame retardant particles were impacted by the crusher blades in the high-speed crusher to make the particles finer. The reduction in particle size is beneficial to the dispersion of the flame retardant in the matrix and the reduction of hole blockage and filament breakage during spinning.

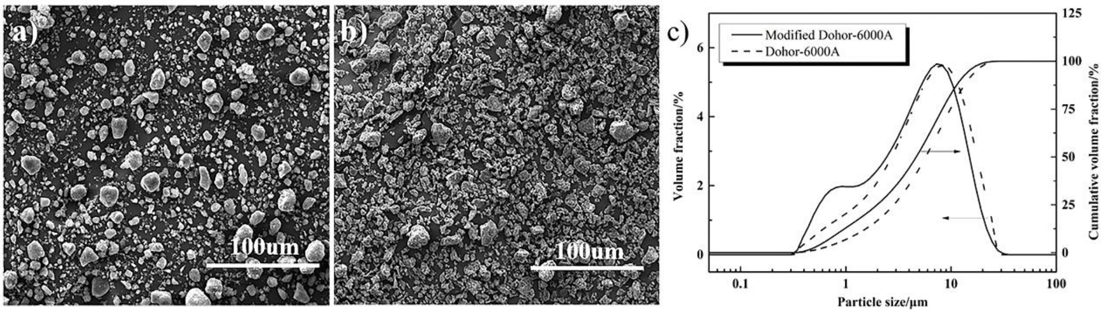

Figure S1. SEM imags of Dohor-6000A (**a**) and modified Dohor-6000A (**b**), the particle size and its distribution of Dohor-6000A and modified Dohor-6000A (**c**).

Figure S2a is the EDS spectrum of the element content on the surface of modified Dohor-6000A particles. It is observed that the surface of the modified flame retardant contents the elements of phosphorus, nitrogen, carbon and silicon. For the unmodified Dohor-6000A has no silicon element, the silicon element within the modified one belongs to KH-550. It proves that KH-550 is successfully attached to the surface of the flame retardant particles. Figure S2b is the distribution diagram of silicon element. It can be observed that most of the flame retardant particles have silicon element on the surface, proving the surface modification in a large scale. It may help the dispersion of the flame retardant in the polypropylene matrix.

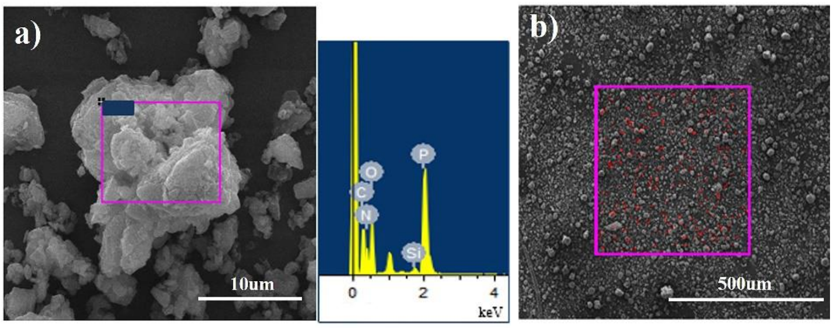

Figure S2. SEM images of modified Dohor-6000A and element of its surface (**a**), the distribution of silicon on the surface of modified Dohor-6000A as detected by EDS (**b**).

Table S1. The particle size of the Dohor-6000A before and after modification.

Sample	Particle size/μm	
	Before modification	After modification
D10%[a]	1.215	0.938
D50%	6.544	5.272
D90%	15.147	13.756
Average	7.787	6.477

[a]The alphabet D represents the percentage of the number of particles in the cumulative curve to the total number of particles.

Table S2. The content of elements on the surface of modified Dohor-6000A.

Element	Weight percentage/%	Atomic percentage/%
N	28.45	33.99
O	54.07	56.54
Si	0.49	0.29
P	16.99	9.18

Table S3. The TG data of a series of fibers under air atmosphere.

Sample	T_0[a]/ ℃	T_{max}/ ℃	T_d/ ℃	Residue/wt%		
				500 °C	600 °C	700 °C
mPP	239.4	349.9	364.8	0.99	0.36	0.07
mPP-IFR-10	262.1	370.5	402.8	10.88	7.19	4.60
mPP-IFR-20	266.9	377.7	415.7	15.43	10.99	7.05
mPP-IFR-25	271.9	376.2	419.6	18.73	14.02	9.22

[a] The T_0 is the temperature of 5 wt% weight loss.

Table S4. Identification of molecule configuration in the evolved products.

Wavenumber/cm^{-1}	Peak/cm^{-1}	Group Vibration	Product
3800-3500	3740	O–H Stretch	H_2O
1580-1480	1506	H–O–H Bending	
3150-2600	2932	C–H Stretch	Hydrocarbon
3150-2600	3016	=C–H Stretch	
1500-1300	1470	C–H Bending	
2400-2200	2360, 2320	C=O Stretch	CO_2
3500-3300	3336	N–H Stretch	NH_3
1650-1580	1626	N–H Bending	
1000-900	965, 930	N–H Bending	
3150-2600	2822, 2726	C–H Stretch	
3800-3500	3740	O–H Stretch	
